# A Mitochondrial Phylogeny and Biogeographical Scenario for Asiatic Water Shrews of the Genus *Chimarrogale*: Implications for Taxonomy and Low-Latitude Migration Routes

**DOI:** 10.1371/journal.pone.0077156

**Published:** 2013-10-04

**Authors:** Shou-Li Yuan, Xue-Long Jiang, Zhen-Ji Li, Kai He, Masashi Harada, Tatsuo Oshida, Liang-Kong Lin

**Affiliations:** 1 Laboratory of Wildlife Ecology, Department of Life Science, Tunghai University, Taichung, Taiwan; 2 State Key Laboratory of Genetic Resources and Evolution, Kunming Institute of Zoology, Chinese Academy of Sciences, Kunming, Yunnan, China; 3 College of the Environment and Ecology, Xiamen University, Xiamen, Fujian, China; 4 State Key Laboratory of Genetic Resources and Evolution, Kunming Institute of Zoology, Chinese Academy of Sciences, Kunming, Yunnan, China; 5 Biological Sciences department, University of Manitoba, Winnipeg, Manitoba, Canada; 6 Laboratory Animal Center, Graduate School of Medicine, Osaka City University, Osaka, Japan; Texas A&M University, United States of America

## Abstract

The six species and three subspecies in the genus *Chimarrogale* (Soricomorpha: Soricidae) are commonly referred to as Asiatic water shrews. The *Chimarrogale* are the most widely distributed group of Nectogaline shrews, extending throughout the Oriental region and Japan. Because of the limited numbers of specimens available for study, the phylogenetic relationships and biogeographical history of this genus have not been comprehensively discussed. We used mitochondrial cytochrome *b* gene sequences to estimate phylogenetic relationships and divergence times among four *Chimarrogale* species, including all three subspecies of *Chimarrogale himalayica*. We also conducted a species delimitation analysis and tested two alternative migration scenarios in Asia through species distribution modeling and a reconstruction of the ancestral distribution. Here, we present the first proposed hypothesis regarding the Asiatic water shrew phylogeny and reveal ten putative species within the four recognized species. Distinct phylogenetic statuses of *Chimarrogale phaeura*, *Chimarrogale platycephala*, and *Chimarrogale styani* were confirmed. *Chimarrogale himalayica* was strongly supported as paraphyletic. We suggest that three subspecies of *Chimarrogale himalayica* should be reconsidered as distinct species. However, these suggestions must be considered with caution because only a single locus of a mtDNA gene was used. Four additional putative species, possibly distributed in central southwestern China and Taiwan, are currently undescribed; therefore, comprehensive morphological analyses are warranted to test their taxonomic statuses. The estimated molecular divergence times indicated that rapid speciation occurred during the early Pliocene, and current distribution patterns may have been affected by global cooling during the Pliocene/Pleistocene boundary. Reconstruction of the ancestral distribution and species distribution modeling for Asiatic water shrews revealed a low-latitude migration route over which ancestral *Chimarrogale* migrated from Europe via Central Asia to their current distribution. Our results demonstrated that Asiatic water shrews could have evolved throughout the low-latitude migration route from Europe to East and Southeast Asia.

## Introduction

Species in the genus Chimarrogale Anderson 1877 are commonly referred to as Asiatic water shrews. These small insectivores belong to the tribe Nectogalini in the family Soricidae and are adapted to a semi-aquatic lifestyle. Based on the latest review by Hutterer [1], six species are recognized in this genus: *Chimarrogale himalayica* Gray, 1842 [2] is distributed in northern India, Nepal, Laos, Myanmar, Vietnam, southwestern and southeastern China, and Taiwan; *Chimarrogale styani* de Witon 1899 is distributed in southwestern China and northwestern Myanmar; *Chimarrogale platycephala* Temmink, 1842 is endemic to Japan; *Chimarrogale phaeura* Thomas, 1898 is distributed in northern Borneo; *Chimarrogale hantu* Harrison, 1958 occurs on the Malaysian Peninsula; and *Chimarrogale sumatrana* Thomas, 1921 is distributed in Sumatra, Indonesia [1,3]. Three additional subspecies have been described within *C. himalayica* by Hoffmann [4]: *C*. *h. himalayica* in the Himalayas; *C*. h. *varennei* Thomas 1927 in Myanmar, Yunnan (China), Laos, and Vietnam; and *C*. *h. leander* Thomas, 1902 in Fujian (China) and Taiwan. Previous classifications of this genus have been based on external morphological and cranial characters and have been in doubt for several years (see Motokawa et al. [5] for the details). Though Motokawa et al. [5] confirmed the valid species status of *C. platycephala* in Japan, they only partially investigated the phylogenetic relationships between Taiwanese and Japanese water shrews. Thus, the phylogenetic relationships and biogeographic history of Asiatic water shrews were left unresolved.

Fossil records suggest that shrews (the Soricidae) originated from Europe in the early Oligocene [6]. Dubey et al. [7] hypothesized, based on a molecular phylogenetic analysis, that members in the subfamily Soricinae had dispersed from west to east Eurasia via a high-latitude route and that these migration and evolutionary events took place in the middle Miocene. Analyzing multi-locus genetic markers for the tribe Nectogalini, He et al. [8] proposed two different migration scenarios: first, the ancestral Nectogalini originated in Europe, then dispersed to Central Asia, and finally to East and Southeast Asia; and second, the ancestral Nectogalini originated in Europe but migrated east to Siberia before dispersing into East and Southeast Asia. The second scenario was considered to be most likely according to fossil records of the Nectogalini and probably occurred in the Miocene, following rapid speciation and southward migration at approximately the Miocene/Pliocene boundary (M/P boundary). He et al. [8] also supported independent phylogenetic origins of semi-aquatic adaptation corresponding to the ancestral stocks of *Neomys* (Eurasian water shrews), *Chimarrogale* and *Nectogale*. If these three genera evolved together following the same migration route, we would expect fossils of their members to be found in similar areas. However, *Chimarrogale* have never been found outside of Asia [6,9]. Current distribution patterns also show that *Neomys* occurs only at high latitudes in Eurasia, while the distribution of *Chimarrogale* is limited to the lower latitudes of East Asia and Southeast Asia [1,10]. Moreover, sister clades to *Chimarrogale*, identified through a phylogenetic analysis by He et al. [8], are distributed only along the southern edge of the Tibetan Plateau and southern China. Therefore, when compared to the history of the high-latitude *Neomys*, these clades might display a more similar evolutionary history to Asiatic water shrews.

*Chimarrogale* is the only Nectogaline genus that is widely distributed throughout the Oriental region and Japan. However, the phylogenetic relationships of its members are poorly understood [5,11]. Because the habitat of Asiatic water shrews is limited to drainage systems, such as rivers or streams in the mountains [10,12], it is easier to track their biogeographic history and test the two migration scenarios proposed by He et al. [8]. Therefore, we employed mitochondrial DNA cytochrome *b* gene (cyt-*b*) sequences and species distribution modeling to examine the phylogenetic relationships and biogeographic history of Asiatic water shrews, focusing on the four main species (*C. himalayica*, *C. phaeura*, *C. platycephala*, and *C. styani*) found in East Asia and Southeast Asia. 

## Materials and Methods

### Ethics statement

We followed the guidelines approved by the American Society of Mammalogists [13] for the capture and handling of animals to minimize harm. Animal samples were collected from China in accordance with implemented regulations for the protection of terrestrial wild animals (State Council Decree [1992] No.13) and were approved by the Ethics Committee of the Kunming Institute of Zoology, Chinese Academy of Sciences, China (no specific permit number). Animal samples from Taiwan were collected with a permit (permit number: 0980137966) according to the guidelines provided by the Council of Agriculture, Taiwan. Other samples were provided from collections at Osaka City University Graduate School of Medicine, Japan, and were approved for use by the collection manager.

### Genetic materials

We obtained 32 mitochondrial cytochrome *b* (cyt-*b*) sequences from four species of Asiatic water shrews (*C. himalayica*, *C. phaeura*, *C. platycephala*, and *C. styani*), including three subspecies of *Chimarrogale himalayica*. Specimens were either collected by researchers in mountainous streams or provided by collaborators (Table S1).

### DNA extraction, amplification, and sequencing

For DNA extraction, liver or muscle tissue was preserved in 99.8% ethanol at room temperature. Total DNA was extracted using a DNA Purification KIT according to the protocol provided by the manufacturer (Epicentre®). Each polymerase chain reaction (PCR) mixture of 50 µl contained approximately 50 ng of genomic DNA, 25 pmol of each primer, and 200 µM dNTP mix in reaction buffer consisting of 10 mM Tris-HCl (pH 8.3), 50 mM KCl, 1.5 mM MgCl_2_, and 2.5 units of *Taq* DNA polymerase. The primers for cyt-*b* were based on the primer pair described by Yuan et al. [14]. Amplifications were performed in a thermal cycler for 35 cycles: denaturation was performed at 94 °C for 1 min, annealing at 52 °C for 1 min, and extension at 72 °C for 2 min. A final extension was conducted at 72 °C for 10 min. The obtained PCR products were loaded into a 1.2% agarose gel, stained with ethidium bromide, and observed under UV light. Approximately 100 ng of the purified PCR products was used for direct sequencing with newly designed sequencing primers specific for Asiatic water shrews with the following sequences: wcytb-f (5'-GAGGACAGATGTCCTTTTGAGGGGC-3') and wcytb-r (5'-TCTGGGTCTCCGAGTAGGTCTGG-3'). Sequencing was performed using the BigDye terminator kit, and sequences were determined directly with an ABI 3730*xl* DNA analyzer (Applied Biosystems®).

### Sequence alignment, phylogenetic analyses, and estimation of divergence time

Full sequences of the mitochondrial DNA (mtDNA) cyt-*b* (1,140 bp) were aligned using the software ClustalW in BIOEDIT version 7.0.9.0 and corrected manually [15,16]. Information on the samples and the corresponding sequence accession numbers are listed in Table S1. Phylogenetic analyses and estimation of divergence times for each clade were performed via the Bayesian phylogenetic approach using BEAST version 1.72 software [17]. To compare topologies with subsequent Bayesian results, a pretest was first performed using the maximum likelihood (ML) method with the software MEGA version 5.03 [18]. The evolutionary model for the ML pretest was chosen with the same software using the Bayesian information criterion [19]. The ML tree searching process was performed via the nearest-neighbor-interchange method, with 1,000 bootstrap replications (BS) [18]. This method was also used by MEGA version 5.03 to preselect the evolutionary model to be used in the BEAST analysis. The evolutionary model for each codon was given independently during BEAST phylogenetic estimation to improve tree support [20]. To obtain the divergence time of each clade, we applied a "relaxed molecular clock" in the BEAST analysis, with soft bond calibration to allow uncertainty in the evolutionary rate and calibration points among clades [21]. This method is especially suitable when using the fossil record for calibration [22]. The calibration points for divergence times used for clades were as follows: First, node T18 (mean=6.63, 95% credible interval=4.81-8.84) from He et al. [8] was used to indicate the divergence time between *Neomys* and other Nectogalini shrews, and we included two *Neomys* species (*Neomys fodiens* and *Neomys anomalus*) as outgroups and set the mean value to 6.63, with a standard deviation of 1.20 in a normal distribution for this calibration point. Second, we used the oldest *Chimarrogale* fossil, from the early Pliocene [23], found in southwestern China as the basal calibration point for all *Chimarrogale* living in China. We set a mean value of 0, an offset of 3.60, and a standard deviation of 0.33 within a lognormal distribution to make the upper 95% credible interval (CI) close to 5.33 for this calibration point. Third, we employed the oldest fossil of the Japanese water shrew (*Chimarrogale platycephala*) on record, which is from the late middle Pleistocene [24]. We set a mean value of 0, an offset of 0.13, and a standard deviation of 0.55 in a lognormal distribution to make the upper CI close to 2.59 for this clade and constrained *C. platycephala* as a monophyletic group. All samples of *Chimarrogale* were also constrained as a monophyletic group (ingroup) in the analysis. The Bayesian MCMC chain was performed with 20 million generations and was sampled every 500 generations. We accepted the final result only when the effective sample size (ESS) was >200. Nodes with posterior probabilities (pp) >0.95 were considered to be statistically supported. The ultrametric tree calculated by BEAST was further used for species delimitation following the methods described in Pons et al. [25]. The number of putative species was identified using a single threshold with the general mixed Yule-coalescent model (GMYC) [26]. This method performed well for single-locus data [26], employing the maximum-likelihood statistics and divergence times within a tree to identify the splitting points from the species to the population level. This analysis was implemented using the SPLITS v2.10 package for the R statistical environment (http://barralab.bio.ic.ac.uk/). The mean genetic distance between clades was also calculated. All results were based on a pairwise analysis among groups using the Kimura 2-parameter (K2P) model in MEGA version 5.03 with 1,000 bootstrap replications [18].

### Species distribution modeling

To examine how past climate change affected the distribution range of Asiatic water shrews, we conducted species distribution modeling to construct the current distribution range of *Chimarrogale himalayica*, the most wildly distributed *Chimarrogale* species in East Asia. Then, we used current distribution parameters to construct past distributions (during the last glacial maximum; LGM) and compared the two distributions. We chose the maximum entropy modeling method to construct species distributions using the software MaxEnt version 3.3.3k [27,28]. This modeling method requires only presence records and has been shown to display good performance and high accuracy, even with small sample sizes [27,29,30]. Thirteen reliable, GPS-positioned sampling localities associated with sources of mtDNA materials were used in the model construction (Table S1). We also employed sampling records from the Kunming Institute of Zoology, Chinese Academy of Sciences, to deduce two collection points from Meigu, Sichuan Province, and one point from Binchuan, Yunnan Province, in China for model construction because DNA samples were not available from these areas. Current Bioclim climate data (in 2.5 arc-minutes resolution) were obtained from the WorldClim website (http://www.worldclim.org/) and included all 19 climate variables. We obtained climate data at the same resolution for past environments (LGM; ~21 ka) from the same website. These data originated from the Paleoclimate Modelling Intercomparison Project, Phase II (PMIP2) [31]. Environmental layers were limited to East Asia and were matched with the GPS positions of sampling locations for constructing the species distribution model in MaxEnt. The model construction process was repeated 50 times. Each time, 20% of localities were randomly selected to evaluate model accuracy. The remaining 80% of localities were used to construct the model. The average, including the mean value of the area under the receiver operating characteristic curve (AUC), was generated as the final result. Because our purpose was to compare changes in suitable habitats for the Asiatic water shrew under different climatic environments, we did not determine any threshold value for the "present". Instead, a probability ranging from 0 to 1 for the suitability of a habitat for each climate environment was shown. The AUC value was also employed to evaluate the validity of the final model, which would indicate the probability of the model correctly predicting the present records. A good model should have an AUC value ranging from 0.9 to 1.0 [30].

### Ancestral distribution analysis

**Figure 1:**
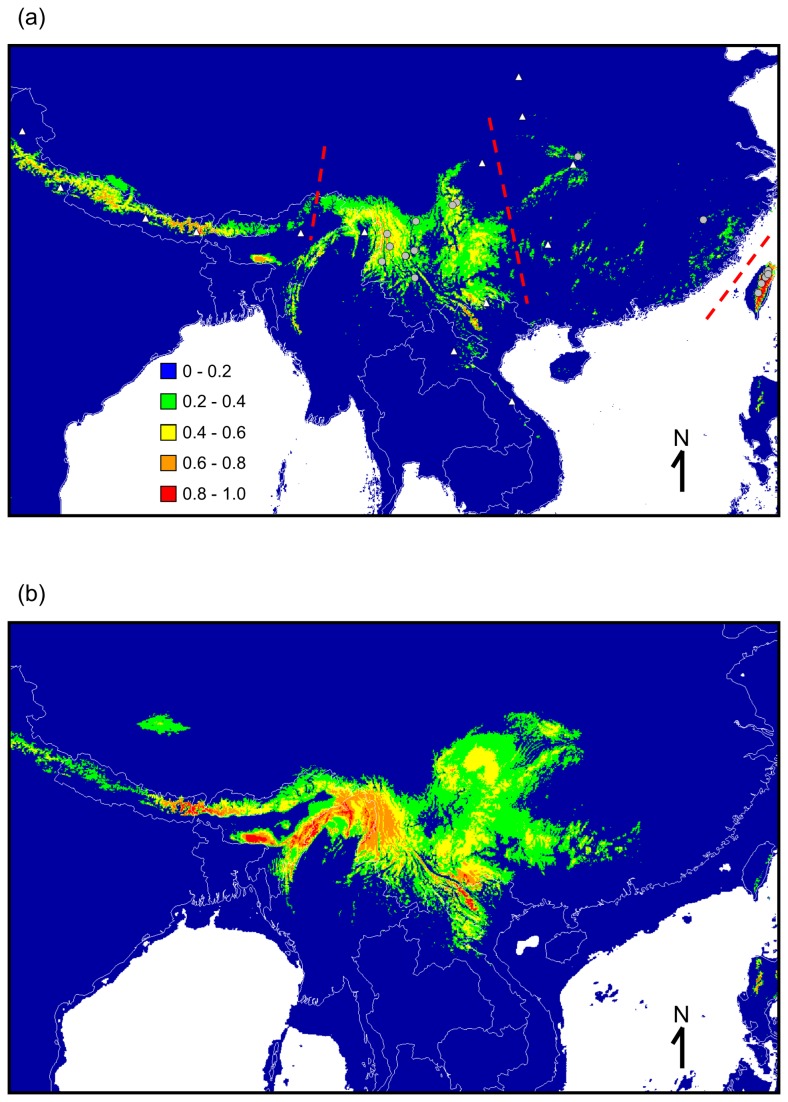
Predicted suitable *Chimarrogale himalayica* habitat based on current (a) and (b) historical climatic conditions. The colors indicate the probability of a habitat being suitable. White indicates areas without climate data. Red dashed lines indicate boundaries for the distributions of the three subspecies of *Chimarrogale himalayica*. Gray circles indicate the localities used to construct the model. White triangles indicate distribution localities summarized by Corbet and Hill [3].

To examine the migration routes of Asiatic water shrews, we used the software RASP version 2.0 (beta) to construct the shrews’ ancestral distribution via Bayesian binary MCMC (BBM) analysis [32], which is available at the web site (http://mnh.scu.edu.cn/soft/blog/RASP). A single majority tree annotated based on Bayesian phylogenetic analysis was used as the basal topology. The geographic region for each taxon was determined to be one of two categories: island or continental. Each island, such as Japan, Taiwan, or Borneo, was considered a separate region. Because *C. himalayica* is distributed across extensive areas of East Asia, each distribution range for the three subspecies in *C. himalayica* was considered a different region (Figure 1a) based on a systematic review by Hoffmann [4]. Seven geographical regions, including outgroups, were determined in the analysis of ancestral distributions: A) *C*. *h. leander* of Taiwan; B) *C. platycephala* of Japan; C) *C*. *h. leander* of eastern China; D) *C*. h. *varennei* and *C. styani* of southwestern China and *C*. h. *varennei* of northern Vietnam; E) *C*. *h. himalayica* of Nepal; F) *C. phaeura* of Borneo; and G) outgroups. The BBM analysis was performed with five million MCMC generations and sampling every 100 generations. The model used in the analysis was a fixed Jukes-Cantor model with a null root distribution. The other settings applied the default values given by the software. 

## Results

### Phylogenetic relationships

**Figure 2:**
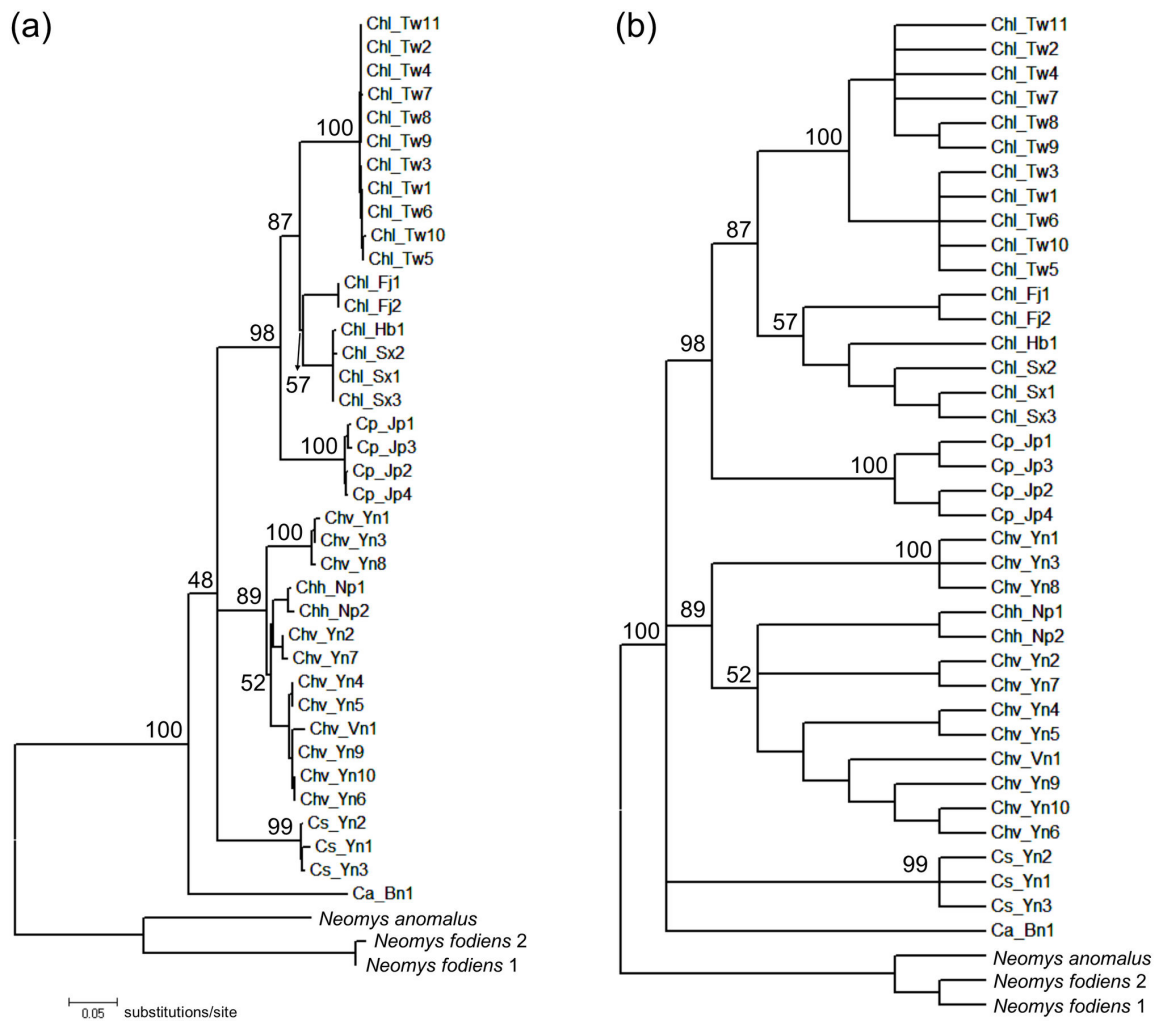
Pretest phylogenetic results for Asiatic water shrews. The results of an analysis of mitochondrial DNA cytochrome *b* sequences via the maximum likelihood method with 1,000 bootstrap replicates are shown. (a) Consensus tree and (b) 50% condensed tree. Numbers on nodes indicate bootstrap support values.

**Figure 3:**
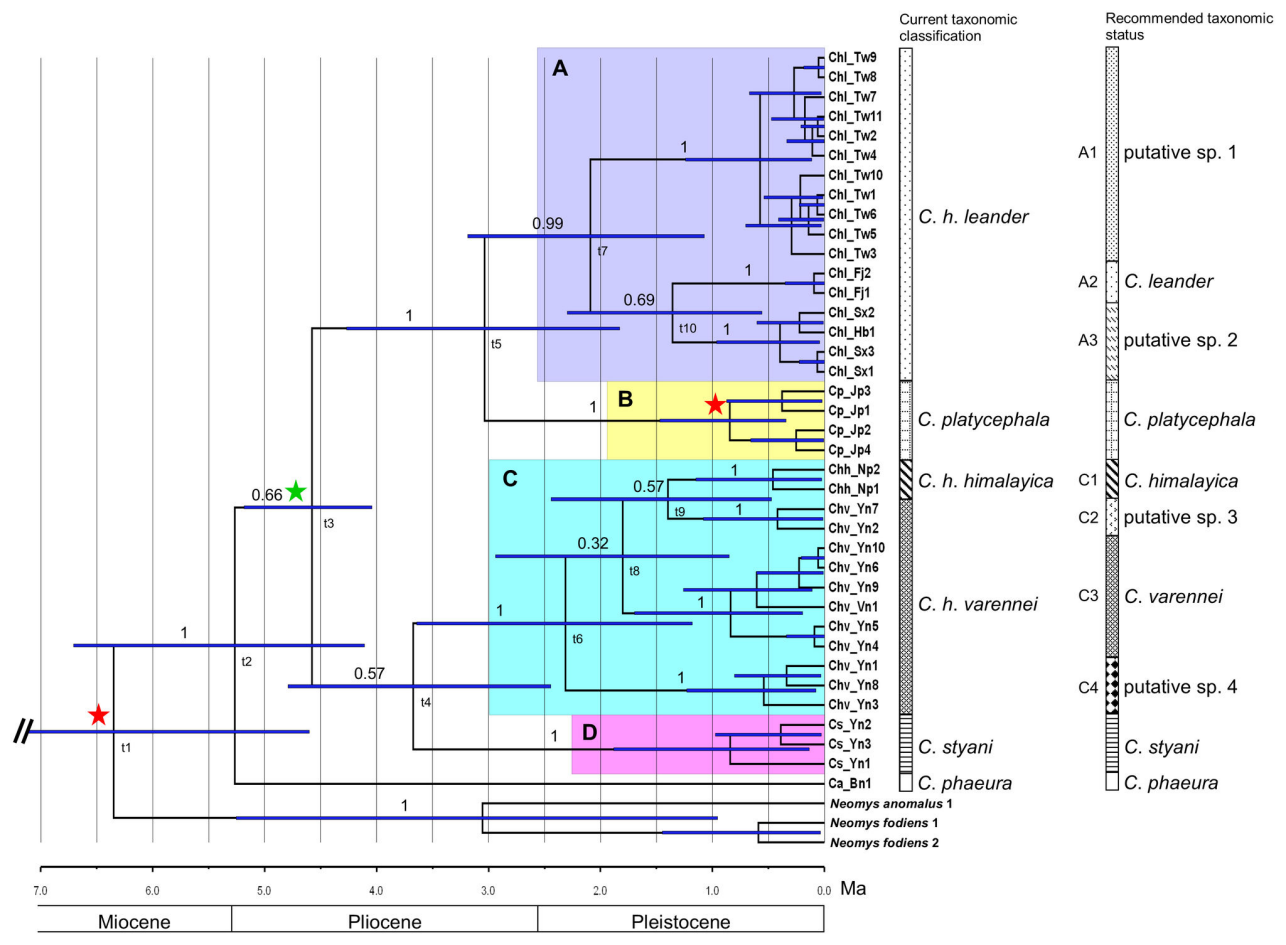
Bayesian phylogenetic results, with divergence time estimations. This is a fossil-calibrated chronogram of a Bayesian phylogenetic analysis, which was constructed from mitochondrial DNA cytochrome *b* sequences, with a relaxed molecular clock. Branch length indicates the time of divergence. Node bars indicate the 95% credible interval for node ages. Numbers above branches indicate the posterior probabilities supporting each node. Letters below the branch nodes indicate the node numbers correlated with Table 4. The four main clades are shaded with different colors (clades A to D). Red stars indicate fossil-calibrated nodes. The green star indicates the calibration for *Chimarrogale* living in China. Correlated Epochs and ages are shown. Current and recommended taxonomic statuses are also shown.

A total of 38 mitochondrial cyt-*b* sequences, including six sequences from GenBank, were analyzed (Table S1). No frameshift mutations or premature stop codons were observed. Detailed information, such as specimen identification numbers and sampling localities, is provided in Table S1. The results of the pretest ML bootstrap consensus tree are shown in Figure 2. A 50% condensed tree (Figure 2b) clarified ambiguous node relationships with lower statistical support. Bayesian phylogenetic results are shown in Figure 3 and were highly concordant with the ML results (cf. Figure 2). The results showed significant support for four monophyletic clades (clade A, B, C, and D; Figure 3) within *Chimarrogale*, while the relationships between them were left unresolved in the ML and Bayesian analyses. *C. phaeura* from Borneo and *C. styani* from Yunnan (clade D, southwestern China) were supported as distinct lineages. *C. platycephala* (clade B) from Japan was well-embedded within *C. himalayica* (BS=98, PP=1.0), indicating the latter as a paraphyletic species (include clades A and C). Three and four subclades were further identified within clades A and C, respectively (Figure 2 and Figure 3). The genetic distances between the four clades and *C. phaeura* are approximately 10.5%-16.4% (Table 1), while those between subclades in clades A and C are approximately 5.7%-8.3% (Table 2) and 3.2%-6.7% (Table 3), respectively.

**Table 1:** Genetic distances among clades of Asiatic water shrews.

**Clade**	**A**	**B**	**C**	**D**	***C. phaeura***
A		0.009	0.010	0.010	0.012
B	0.105		0.011	0.011	0.013
C	0.141	0.141		0.009	0.012
D	0.138	0.138	0.121		0.013
*C. phaeura*	0.163	0.164	0.157	0.153	

Mean genetic distances (lower triangle) and standard errors (upper triangle) among clades based on mitochondrial DNA cytochrome *b* gene sequences determined using a Kimura 2-parameter model with 1,000 bootstrap replications. The names of the clades are provided in Figure 3.

**Table 2:** Genetic distances among the three subclades of Asiatic water shrews within clade A.

**Subclade**	**A1**	**A2**	**A3**
A1		0.008	0.009
A2	0.071		0.007
A3	0.083	0.057	

Mean genetic distances (lower triangle) and standard errors (upper triangle) among the three subclades of clade A based on mitochondrial DNA cytochrome *b* gene sequences determined using a Kimura 2-parameter model with 1,000 bootstrap replications. The names of the subclades are provided in Figure 3.

**Table 3:** Genetic distances among the four subclades of Asiatic water shrews within clade C.

**Subclade**	**C1**	**C2**	**C3**	**C4**
C1		0.005	0.006	0.007
C2	0.032		0.006	0.008
C3	0.040	0.042		0.008
C4	0.065	0.064	0.067	

Mean genetic distances (lower triangle) and standard errors (upper triangle) among the four subclades of clade C based on mitochondrial DNA cytochrome *b* gene sequences determined using the Kimura 2-parameter model with 1,000 bootstrap replications. The names of subclades are shown in Figure 3.

### Molecular divergence times and species delimitation

The divergence time between *Neomys* and *Chimarrogale* was approximately 6.35 Ma (node t1, 95% CI=4.6-8.12 Ma). This value represents a consistent time estimation and is very close to the calculated value reported by He et al. [8] that we used as the calibration point at the root position (Figure 3, Table 4). *Chimarrogale phaeura* was the species that diverged earliest, likely around the Miocene/Pliocene boundary (node t2, mean=5.27, 95% CI=4.11-6.71 Ma). The low pp support at nodes t3 and t4 might indicate rapid evolution at the Miocene/Pliocene boundary and the appearance of *C. himalayica*, *C. phaeura*, and *C. styani* in the same period. The divergence of *C*. *h. leander* and *C. platycephala* occurred at approximately 3.03 Ma (node t5, 95% CI=1.83-4.27 Ma). One distinct subclade (C4) diverged within clade C, which consisted the divergence of *C*. *h. himalayica* and *C*. h. *varennei*, during the same time period (node t6, mean=2.31, 95% CI=1.18-3.64 Ma) in Yunnan Province of China. The divergence of *C*. *h. leander* of Taiwan from the group in eastern China occurred at approximately 2.09 Ma (node t7, mean=5.27, 95% CI=1.07-3.19). These divergence events at nodes t5, t6, and t7 took place around the Pliocene/Pleistocene boundary (Figure 3).

**Table 4:** Divergence times for 10 major nodes.

**Node**	**Mean age (Ma)**	**95% CI (Ma**)
t1	6.35	4.6-8.12
t2	5.27	4.11-6.71
t3	4.58	4.04-5.18
t4	3.67	2.45-4.79
t5	3.03	1.83-4.27
t6	2.31	1.18-3.64
t7	2.09	1.07-3.19
t8	1.8	0.85-2.94
t9	1.4	0.47-2.44
t10	1.36	0.56-2.3

The mean divergence times and 95% credible intervals based on mitochondrial DNA cytochrome *b* sequences determined via Bayesian phylogenetic analysis with a relaxed molecular clock and 10 major nodes. The node names are provided in Figure 3.

The results of the species delimitation analysis revealed clades with divergence times earlier than 0.87 Ma, which were considered to be putative species (n=10), and all clades and subclades shown in Figure 3 were recognized as putative species via the GMYC method.

### Species distribution modeling and ancestral distributions

The results of the suitable habitat prediction performed for *C. himalayica* under current climate variables are shown in Figure 1a. This map showed that our estimation mostly fit the occurrence records for *C. himalayica*. The AUC value of our model was 0.981, indicating very good model performance. Temperature-related variables such as the mean temperature of the coldest month, maximum temperature of the warmest month, and annual temperature range, made the largest contributions to the model (28.2%, 27%, and 17%, respectively). A suitable habitat for *C. himalayica* in the past is shown in Figure 1b. The probability of suitable habitat for *C. himalayica*, which was much more connected between eastern and western areas of East Asia, increased during the glacial period (LGM).

**Figure 4:**
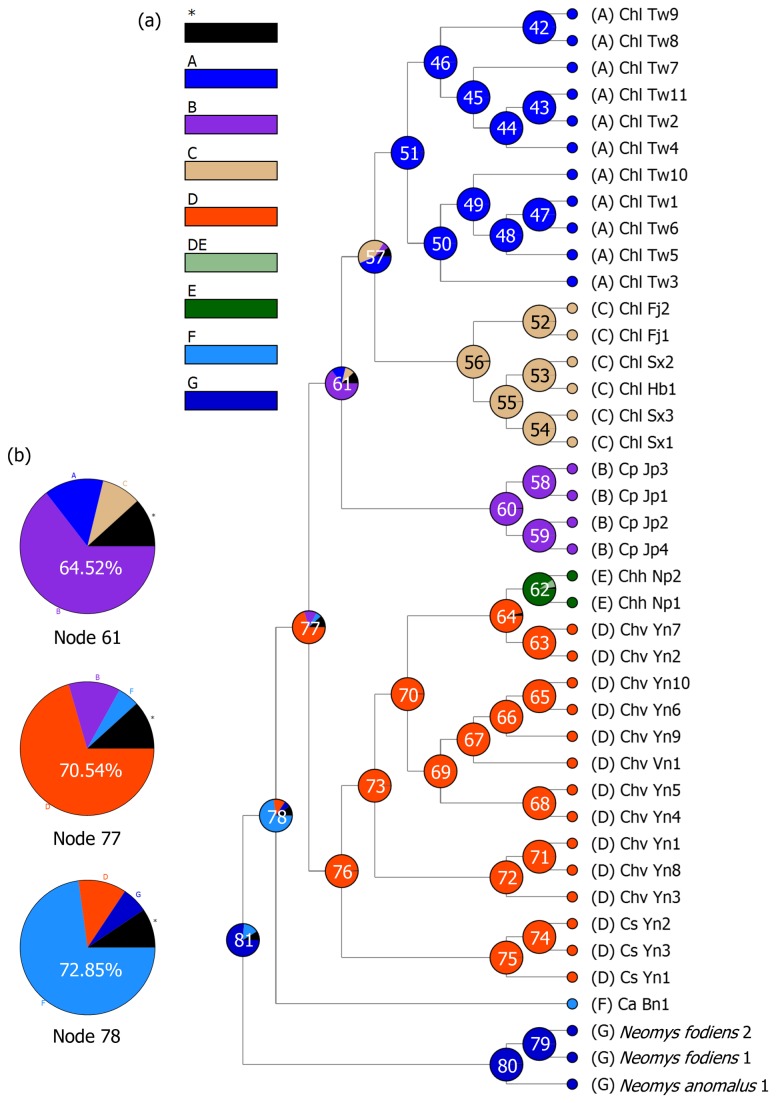
Reconstruction of the ancestral distribution of Asiatic water shrews. Ancestral distribution range based on the Bayesian binary MCMC method. The distribution range is based on the majority tree annotated from Bayesian phylogenetic analysis (a). The classifications of the geographical regions of current samples are identified in different colors, with the letters beside the sample names indicating the following specimens: A) *Chimarrogale himalayica leander* from Taiwan; B) *Chimarrogale platycephala* from Japan; C) *Chimarrogale himalayica leander* from eastern China; D) *Chimarrogale himalayica* *varennei* and *Chimarrogale styani* from southwestern China and *Chimarrogale himalayica* *varennei* from northern Vietnam; E) *Chimarrogale himalayica himalayica* from Nepal; F) *Chimarrogale phaeura* from Borneo; and G) outgroups. The pies indicate the ancestral distribution based on the probabilities (%) at each node. Black stars show the combined probabilities of the reconstruction results <5%. The probabilities (%) of the reconstruction results of three major nodes are shown in (b). Vicariance and dispersal events are indicated at these nodes.

The results of the ancestral distribution analysis performed via BBM are shown in Figure 4a. The origin of *Chimarrogale* might have been in Southeast Asia (node 78), with probabilities of 72.85% for region F and 11.48% for region D being obtained. Furthermore, the origin of *Chimarrogale* in East Asia likely occurred in southwestern China (node 77), with probabilities of 70.54% for region D and 12.52% for region B being observed. The ancestor of the *Chimarrogale* found in eastern China, Taiwan, and Japan potentially occurred in the area around eastern China and Japan, as indicated by a probability of 64.52% for region B (node 61), though there was also a probability of 14.26% that the ancestor originated in region A (Figure 4b). Vicariance and dispersal events were indicated for the nodes mentioned here in the BBM analysis as well. 

## Discussion

### The limitations of mitochondrial DNA

The utilization of mtDNA as a marker in phylogenetic and phylogeographic studies and the associated problems have been well-discussed in the literature (e.g., [33,34]). Because of its rapid evolutionary rate, mtDNA usually can reflect phylogenetic and phylogeographic histories well at both the intra- and interspecific levels [35]. The mitochondrial genome has also been used as a bar code in eutherian mammals [36]. However, increasing evidence suggests that due to heteroplasmy, introgression, and non-neutrality of mtDNA genomic evolution [33,37], mitochondrial gene trees might differ from species trees [38]. Therefore, our results should be treated with caution and tested in future studies using multiple nuclear genes. Nevertheless, this study provides the first-ever phylogeny of the genus *Chimarrogale*, and the results revealed significant patterns that are highly associated with geographic distributions and the paleoclimate.

### Molecular phylogenetics and systematics of *Chimarrogale* species in East Asia

Our phylogeny statistically supports *C. himalayica* as a paraphyletic species. The species-level paraphyly observed in the mtDNA phylogeny commonly occurs in many taxa for various reasons [38]; however, because of the strong phylogeographic pattern, we believe this result occurred due to the existence of cryptic species within the *Chimarrogale*. The results of species delimitation using the GYMC method recognized 10 putative species, but because the analysis was based on a single maternal gene, these results should be reexamined via approaches involving multiple loci [39]. Nevertheless, the taxonomic diversity of *Chimarrogale* has been highly underestimated prior to this study. While future comprehensive morphological diagnoses are needed in future studies, we present a working hypothesis and some preliminary discussion about the taxonomy based on the current results of phylogenetic and species delimitation.

The monophyly of *C. styani* in southwestern China is strongly supported (clade D, pp=1.0; Figure 3), even though its phylogenetic position is largely unresolved. This species exhibits a light-colored ventral surface, which makes it the most morphologically distinguishable of the Asiatic water shrew species [1,4]. Although this species is sympatric with *C. himalayica* [12], the two shrews are highly genetically diverged (K2P distance >12%). Based on the above reasons, *C. styani* was confirmed as a distinct species (Figure 3, Table 1).

*C*. *h. leander*, represented by clade A, was found to be closely related to *C. platycephala* with high statistical support (node t5, pp=1.0; Figure 3). This result supports the findings of Ohdachi et al. [40], who investigated the phylogenetic relationships of *Chimarrogale* based on samples from Japan (*C. platycephala*) and Taiwan (identified as *C. himalayica* in their manuscript). Because the phylogenetic position and taxonomic status of *C. platycephala* of Japan are agreed upon in most studies [1,3,5], Ohdachi et al. [40] proposed that either species status or subspecies status, under *C. platycephala*, be applied to the *Chimarrogale* of Taiwan. However, their analyses did not include the original sampling locality of *C*. *h. leander* from Fujian or any other site in China. Our analysis included two *Chimarrogale* specimens collected from the type locality of *C*. *h. leander* at Wuyishan in Fujian Province in China [41], one specimen from Hubei Province in China, three specimens from Shaanxi Province in China, and 11 specimens from Taiwan. Our results strongly supported the genetic isolation of *C*. *h. leander* from central and eastern China and Taiwan from other *C. himalayica* species (clade A, pp=0.99; Figure 3). The mean genetic distances between *C*. *h. leander* and the other clades were >10.5% in K2P (Table 1). A morphological study showed that specimens of *C*. *h. leander* from southern China and Taiwan presented smaller body sizes [4]. Therefore, we suggest that *Chimarrogale leander* Thomas, 1902 is a valid species rather than a subspecies of *C. himalayica*. The distribution range of *C. leander* included southeastern China and Taiwan [4,5,41].

Motokawa et al. [5] found that *C. platycephala* and *C. phaeura* are phylogenetically distinguishable. We confirmed this result and showed that *C. phaeura* represented a sister group to the other *Chimarrogale* species and subspecies (Figure 3). The mean genetic distance between *C. phaeura* and other clades was >15% in K2P (Table 1). This result was expected because *C. phaeura* is distributed in a lower-latitude area, far from the other *Chimarrogale*, indicating that it may have diverged quite some time ago. Moreover, the differences in the shapes of the upper incisors of *C. himalayica* and *C. phaeura* confirm the distinct taxonomic status of *C. phaeura* [3].

Although morphological investigation did not reveal any significant differences among the three *C. himalayica* subspecies, the Taiwanese water shrew was found to be slightly smaller than the other subspecies [5]. This observation was reported previously by Jones and Mumford [42], who measured the skulls of *Chimarrogale* from Fujian and Taiwan. They recommended subspecies status for *Chimarrogale* in Taiwan. Our results place *C*. *h. leander* of Taiwan in a monophyletic group with high statistical support (subclade A1, pp=1.0; Figure 3). The mean genetic distance between the populations from Fujian and Taiwan was 7.1% in K2P (Table 2), and they diverged at approximately 2.09 Ma (node t7, Table 4). This result is nearly identical to the divergence time observed between *Chodsigoa parca* of Yunnan and *Chodsigoa sodalis* of Taiwan, as described by He et al. [8], which might indicate the existence of similar divergence events driving the speciation of these small mammals. In summary, due to the observed geographical isolation and genetic diversification, we support the findings of Ohdachi et al. [40] and suggest that *C*. *h. leander* from Taiwan might represent a cryptic species (Figure 3).

The samples collected from Hubei and Shaanxi provinces of China were recognized as belonging to subclade A3 (Figure 3). The genetic distance between subclades A2 and A3 was 5.7% in K2P. Based on the species delimitation analysis, a putative species status was indicated for this subclade. Due to a lack of sufficient specimens and of knowledge of the distribution range, we temporarily considered this putative species to be located in central China.

Samples from Trans-Himalaya regions, including *C*. *h. himalayica* from Nepal and *C. h. varennei* from Vietnam and Yunnan Province in China, were clustered together into clade C (pp=1.0; Figure 3). Unexpectedly high genetic polymorphism was observed, and four subclades were statistically supported (subclades C1 - C4, pp=1.0; Figure 3). Hoffmann [4] concluded that the *Chimarrogale* distributed in the southern Himalayas should be classified as *C*. *h. himalayica* and that those distributed in southwestern China and Vietnam should be classified as *C. h. varennei*. Our two samples from Nepal were constrained into subclade C1, which supports the suggestions made by Hoffmann [4], and these samples could be classified as *C*. *h. himalayica*. However, the samples from southwestern China and Vietnam clustered into three different subclades and showed a paraphyletic relationship with *C*. *h. himalayica* within clade C (subclades C2, C3, and C4; Figure 3). This result does not support Hoffmann [4] classification of *C. h. varennei*. Subclade C2 was most closely related to subclade C1 and included two samples from Yunnan Province, China. Five samples from Yunnan Province and one sample from Vietnam were grouped into another subclade with strong statistical support (subclade C3, pp=1.0). The type locality of *C. h. varennei* Thomas, 1927 is located in Dakto, in central Vietnam [3]. Therefore, subclade C3 most likely corresponded to *C. h. varennei*. Subclade C4 was the most distinct subclade within clade C, and the genetic distances between C4 and the other subclades were >6% in K2P (Table 3). The results of species delimitation using the GYMC method indicated that these four subclades within clade C are putative species. Because there were insufficient available samples and we did not know the distribution ranges of subclades C2 and C4, we tentatively labeled these subclades with an unknown putative species status. Under the same criterion used in the GYMC method, the two current subspecies of *C. himalayica* (subclade C1 and C3) would also be considered valid species (Figure 3). However, as emphasized above, these suggestions must be considered with caution because they were based on a very limited sample size, and only a single locus of a mtDNA gene was used. Comprehensive sampling in southwestern China or examination of museum specimens using next-generation sequencing methods will be necessary to clarify the phylogenetic relationships in this area [43].

### Biogeography, paleoclimate, and an evolutionary scenario for Asiatic water shrews

Although the population sizes of *Chimarrogale* are relatively low compared to those of other shrews [5,11], their distribution extends throughout the entire Oriental region. This makes *Chimarrogale* the most widely distributed genus of the Asian Nectogalini and enables us to examine the biogeographic history of Nectogalini shrews in Asia. Although He et al. [8] proposed a high-latitude migration route for the Nectogalini, we found evidence supporting a low-latitude route that better explains the observed current distribution patterns and the phylogenetic structure of *Chimarrogale*.

The relationship between the paleoclimate and mammalian evolution has been well documented [44-46]. Similar to other mammals, the migration and distribution of *Chimarrogale* were strongly affected by global climate change during the Neogene and Quaternary. Species distribution modeling showed that the suitable habitat for *Chimarrogale* expanded during the LGM (Figure 1b). Temperature-related variables made the greatest contribution to the model’s construction, further indicating that the suitable habitat for *Chimarrogale* increased during the glacial period. Although climate data from prior to the LGM were not available, our results indicated that *Chimarrogale* populations might have increased during glacial periods and decreased during interglacial periods. This result is consistent with data obtained from field surveys, as Asiatic water shrews can currently only be found in mountain streams and rivers [10,11,47].

The ancestral distribution analysis conducted via BBM indicated that the possible locality of the *Chimarrogale* common ancestor showed a 72.85% probability of being situated in Southeast Asia (node 78; Figure 4). Our BBM results also suggested that the ancestral localities of clades A+B, C, and D may have been in southwestern China (70.54% probability, node 77; Figure 4). *Chimarrogale* species most likely migrated from Europe to Asia via a low-latitude route, such as through Central Asia.

**Figure 5:**
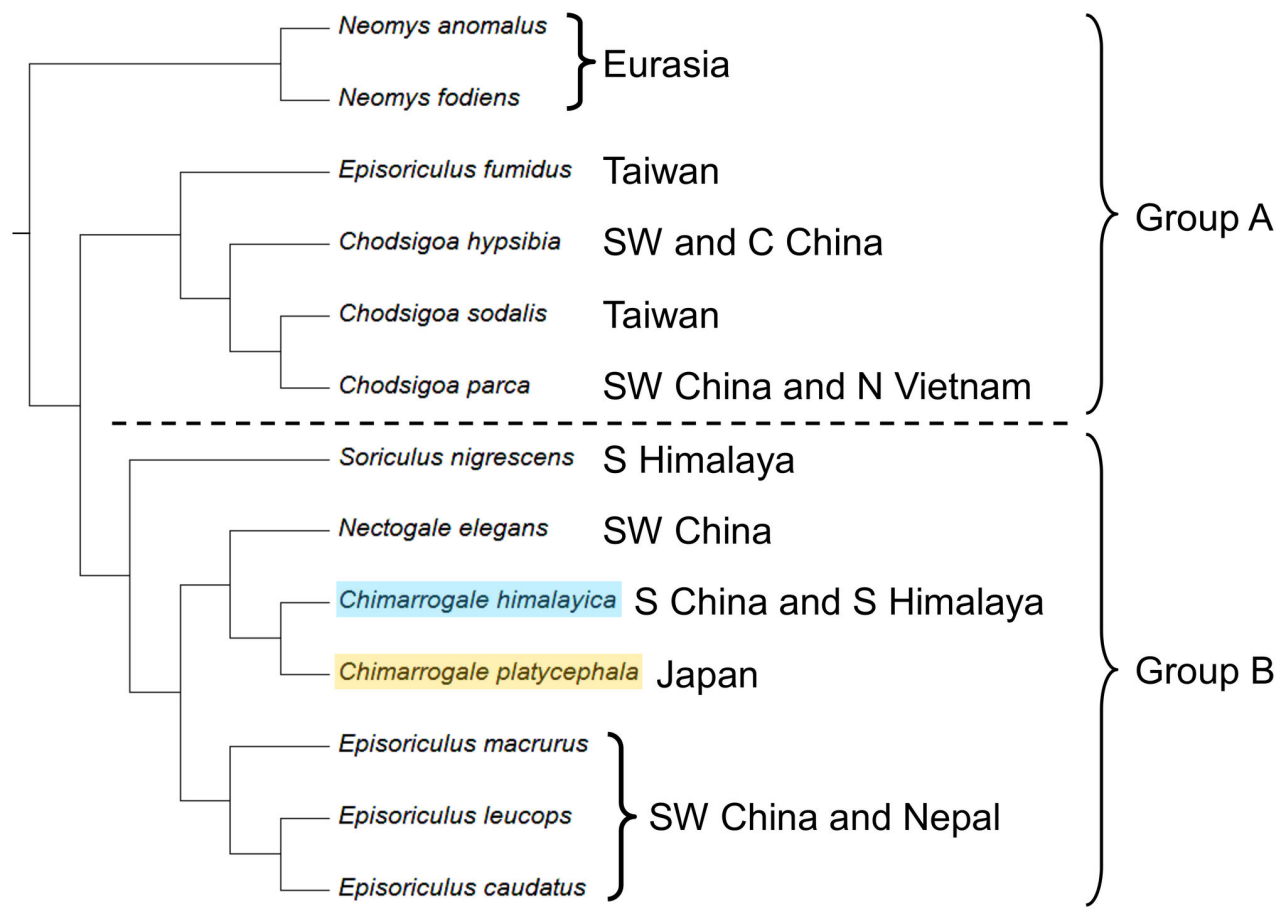
Molecular phylogenetic results modified from He et al. **[8]**. The topology of the Bayesian phylogenetic results obtained by combining ApoB (615 bp) and BRCA1 (792 bp) gene sequences modified from He et al. [8]. The distribution range for each species is based on the Red List of the International Union for Conservation of Nature and Natural Resources, accessible online at (http://www.iucnredlist.org/). The dotted line divides groups A and B according to geographical regions.

Fossil records [6,9] and molecular phylogenetic analyses [7] indicate that shrews originated in Europe in the early Oligocene and dispersed to Asia during the middle Miocene. He et al. [8] concluded that the Nectogalini fossils found in northern Asia are more primitive and older in age than those from southern Asia, indicating a southward migration route. They also suggested that Nectogalini shrews migrated to lower latitudes around the M/P boundary and that *Chimarrogale* dispersed throughout the Chinese region after the Pliocene/Pleistocene (P/P) boundary. When considering only semi-aquatic shrews, if *Chimarrogale* and *Neomys* evolved and migrated together from northern to southern Eurasia, then the question remains of why *Chimarrogale* fossils are only found in central and southern China [9], whereas *Neomys* fossils are only found at high latitudes in Eurasia [6]. The fossil record indicates that *Chimarrogale* had already appeared in central China in the early Pliocene [23]. Our model further indicates that *C. phaeura* from Borneo diverged from the other species and subspecies at approximately the M/P boundary (Figure 3), implying that *Chimarrogale* arrived in Southeast Asia during the late Miocene. Finally, one fossil of *Soriculus* (a member of tribe Nectogalini) from late Pliocene strata was found in Kashmir, in northwestern India [9]. The occurrence of this fossil cannot be explained by a high-latitude scenario. In Figure 5, we present a tree topology modified from that of He et al. [8], which shows that the distribution ranges of group A and group B were clearly different. The species in group A mainly occurred in central China and high-latitude Eurasia (*Neomys*), while those in group B are mainly from southwestern China and the southern Himalayas. These different distributions might indicate divergent migration or evolution scenarios. High-latitude migration and evolution explained the distribution of group A well but not that of group B.

**Figure 6:**
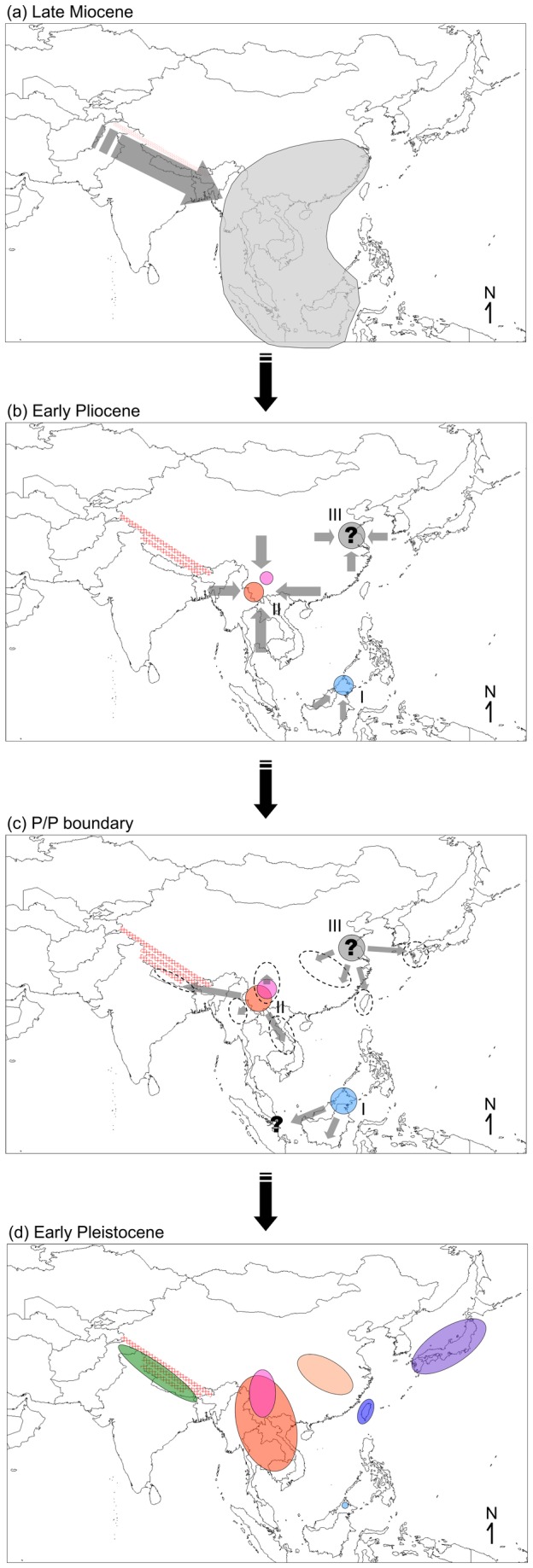
Hypothetical biogeographic history of Asiatic water shrews. Ancestral distribution of Asiatic water shrews (in shaded gray color) during the late Miocene in East and Southeast Asia (a). The population decreased during warming in the early Pliocene (b). The population expanded and re-colonized surrounding areas during the glaciation that occurred at the Pliocene/Pleistocene boundary (c). The population decreased again during warming in the early Pleistocene. The colors indicate corresponding geographical regions shown in Figure 4. The distribution range of *Chimarrogale styani* is shown in pink (d). Population increases and decreases are indicated by arrows. Roman numerals indicate the three possible refuges. Question marks indicate an unknown refuge area in East Asia. The ranges of the southwestern Tibetan Plateau are shown in bold red lines.

Based on our results, we hypothesized that an ancestor of *Chimarrogale* was mainly distributed in East and Southeast Asia during the late Miocene, after migrating from Europe through Central Asia. This hypothesis also supports independent evolutionary origins for semi-aquatic adaptations in *Chimarrogale* and *Neomys* in He et al. [8]. If Asiatic water shrews followed this low-latitude migration and evolution scenario, then their biogeographic history would have been similar to that shown in Figure 6. The predicted divergence times indicated that the first divergence event of the ancestral *Chimarrogale* was near the M/P boundary (node t2, 5.27 Ma; Table 4). The short branches and low node supports observed in the ML pretest tree and Bayesian tree suggest that rapid speciation occurred after the M/P boundary, during which clades A+B, C, D, and *C. phaeura* might have diverged (Figures 2, 3). Global cooling and drying events occurred gradually after the middle Miocene [48,49], when the *Chimarrogale* ancestor would have expanded from Europe to Asia, as this species was adapted to cool environments (Figure 6a). Finally, climate change in the late Miocene induced changes in global vegetation and habitat that caused turnover and rapid speciation of global fauna around the M/P boundary [50-52]. Rapid speciation phenomena during this time have been noted for Nectogaline shrews [8] and other mammals [53-55]. Similar to the other Nectogaline shrews, habitat changes might have reduced the potential habitats available for *Chimarrogale* species. Global warming during the early Pliocene [56-58] may have driven rapid speciation of the genus because of the low tolerance of its members to higher temperatures. Around the M/P boundary, the ancestral population of *Chimarrogale* might have decreased, taking refuge in some areas in which rapid speciation could have occurred (Figure 6b). Vicariance and dispersal events indicated by the BBM analysis of nodes 61, 77, and 78 support the notion that speciation took place via radiation during this time (Figure 4). Therefore, we propose three possible refugia for *Chimarrogale*: northern Borneo (I); southeastern China (Hengduan Mountains; II); and an unknown locality in eastern China (III; Figure 6b).

Regarding refuge I, geographic studies indicate that the highlands of Sundaland were elevated in the middle Miocene, connecting the western side of Wallace’s Line with mainland Southeast Asia until the late Pliocene [59,60]. Therefore, it was possible for *Chimarrogale* to be distributed throughout this region, before taking refuge in the highlands of northern Borneo. Similar divergence events have been reported for the early Pliocene, as demonstrated by phylogenetic studies of *Crocidura* shrews [61,62] and murine rodents [63] in the Sundaland region.

Concerning refuge II, ecological and biogeographical studies show that the complex environment of the Hengduan Mountains in southwestern China and northern Vietnam (the southeastern Tibetan Plateau) were important for conserving biodiversity and inducing speciation in mammals [64-66]. Geographic evidence suggests that the southeastern area of the Tibetan Plateau was elevated during the middle Miocene [67]. During the global warming that took place in the early Pliocene, the *Chimarrogale* population in south and southwestern Asia most likely became extinct, except for the shrews within a refuge in the southeastern area of the Tibetan Plateau. The uplift of the Tibetan Plateau could have trapped moisture [68], providing a cool and humid habitat for *Chimarrogale*. *Chimarrogale himalayica* and *C. styani* most likely appeared during this period but might have originated in different ranges of the Hengduan Mountains. This is concordant with the earliest fossil record of *Chimarrogale*, which was found in southwestern China and was dated to the early Pliocene [23]. Our refuge hypothesis is consistent with the divergence and speciation of other fauna on the Tibetan Plateau, which mostly occurred during the early Pliocene to late Miocene [69].

With respect to refuge III, ancestral analysis via BBM indicated that the possible ancestral distribution range of clade A+B was located in Japan (64.52%), but there was an almost 36% probability of it being located in Fujian, Taiwan, or elsewhere (node 61, Figure 4). Therefore, we propose that there was an unknown refuge within East Asia, most likely in eastern China. This hypothesized refuge was also noted in a biogeographic study on the *Crocidura suaveolens* group conducted by Dubey et al. [70], who proposed the existence of a refuge on the Pacific coast. We consider the eastern side of the Chinese Loess Plateau to be more suitable for Asiatic water shrews because geographic evidence suggests that this area became arid approximately 6 Ma but was more humid in the early Pliocene [71,72]. A cool and humid regional climate would have provided suitable shelter for *Chimarrogale* during this time.

Figure 3 shows that a second divergence event began around the P/P boundary, during which the divergence of clade A+B (node t5, 3.03 Ma), within clade C (t6, 2.31 Ma), and within clade A (t7, 2.09 Ma; Table 4) occurred. Similar to the M/P boundary, rapid speciation most likely occurred during this age, especially within clade C (which was supported in our ML pretest tree; Figure 2). Global cooling occurred during the late Pliocene and the P/P boundary [56,73]. In Europe, some mammals retreated southward during the glacial periods of the Quaternary and expanded north again during inter-glacial periods [46,74]. As *Chimarrogale* have occurred in the lower latitudes of Asia since the early Pliocene, we propose that global cooling around the P/P boundary provided a good opportunity for Asiatic water shrew populations to expand from the three refuges of the early Pliocene (Figure 6c). Shrews in refuge I most likely colonized the Malaysian peninsula and Sumatra, affecting the current distribution range of *C. hantu* and *C. sumatrana*. Although we did not analyze any genetic material from these two species in the present study, the similarity of the morphology of the upper incisors of *C. hantu*, *C. phaeura*, and *C. sumatrana* indicates that these species evolved from a common ancestor [4]. We propose that the speciation of these three shrews occurred in the same age, which was most likely after the P/P boundary. This hypothesis might be supported by examining the phylogenetic relationships of two endangered *Chimarrogale* species, *C. sumatrana*, and *C. hantu*.

In refuge II, which includes the re-colonization of Myanmar, Vietnam, and the southern and southeastern Tibetan Plateau, species distribution modeling indicated that the suitable habitat for Asiatic water shrews between southwestern China and southern Tibetan Plateau was largely connected during the cold period (Figure 1b). As the Tibetan Plateau was already at its current elevation during the early Pleistocene [75], colonization from a refuge in the Hengduan Mountains to the southern Tibetan Plateau was possible. These mountainous regions would have provided suitable habitat for those re-colonized *Chimarrogale* populations during later interglacial periods. The fossil record of *C. himalayica* in southwestern China from the early Pleistocene [9] also supports this hypothesis.

For refuge III, colonization from eastern China to coastal areas, Japan, and Taiwan is concordant with a phylogeographic study on Japanese water shrews, which suggested that the Japanese population originated from Kyushu (southwestern Japan), before dispersing throughout the island of Japan [76]. The fossil record also indicates that *C. platycephala* appeared before the middle Pleistocene [24], which also supports our colonization hypothesis.

After the cold period of the P/P boundary, speciation occurred during the following interglacial period of the early Pleistocene. Populations decreased again during this warm period [77] to form the present distribution pattern (Figure 6d). Subsequent climate oscillation in the late Quaternary might have affected the genetic structure of *Chimarrogale*, but additional samples will be required to clarify the phylogeographic relationship between *Chimarrogale* populations and climate changes in East and Southeast Asia. 

## Supporting Information

Table S1 **Information of samples used in present study.** Sampling locality, museum collection code, and GenBank accession number of each sequence is provided. Corresponding species names and regional codes for Bayesian binary MCMC analysis are also shown in this table.
(XLSX)
